# PTGER4 Governs Immune Evasion and Therapy Resistance in Kidney Cancer via Ribosome Biogenesis Dysregulation

**DOI:** 10.1111/jcmm.70956

**Published:** 2025-11-24

**Authors:** Hanjing Zhou, Zirui Li, Jun Ying, Yan Liu, Xuchun Xu, Jian Huang

**Affiliations:** ^1^ Department of Nephrology Jinhua Hospital of Zhejiang University Jinhua China; ^2^ Center for Cancer Bioinformatics Peking University Cancer Hospital & Institute Beijing China

**Keywords:** immunotherapy, KIRC, machine learning, PTGER4, ribosome biogenesis

## Abstract

Kidney renal clear cell carcinoma (KIRC) is associated with abnormal ribosome production (RiboSis), but how this affects tumour growth and response to immunotherapy is still unclear. In this study, we analysed large‐scale multi‐omics data using machine learning. Using single‐cell RNA sequencing and gene network analysis (hdWGCNA), we found a key RiboSis‐related gene group. We then classified KIRC tumours into two subtypes based on RiboSis activity. Patients with subtype 1 lived significantly longer, and this group showed activation of tumour‐promoting pathways. Using machine learning, we identified PTGER4 as a potential tumour suppressor. Higher PTGER4 levels were linked to better survival in multiple patient groups. Tumours with high PTGER4 also had stronger immune cell activity and higher levels of immunotherapy‐related markers, suggesting they may respond better to immune‐based treatments. PTGER4 also predicted better outcomes with certain chemotherapy drugs. Further analysis confirmed that PTGER4 is involved in immune‐related pathways and is often reduced in tumours, supporting its role in slowing cancer progression. Lab experiments confirmed that PTGER4 helps block tumour growth. These findings suggest PTGER4 plays a central role in KIRC progression and treatment response. Targeting RiboSis‐related mechanisms and PTGER4‐related pathways could lead to better therapies for KIRC patients.

## 1 Introduction

Kidney renal clear cell carcinoma (KIRC) is a malignancy marked by metabolic reprogramming and immune evasion, contributing to its resistance to conventional therapies [1]. Ribosome biogenesis (RiboSis), a hallmark of cancer, drives tumour proliferation and adaptation [2], yet its role in KIRC pathogenesis and immune modulation remains poorly understood. Recent studies implicate RiboSis‐related genes in shaping the tumour microenvironment (TME) and influencing clinical outcomes [3], but their prognostic and therapeutic relevance in KIRC warrants systematic investigation.

The TME of KIRC comprises heterogeneous cell populations, including immune, stromal, and malignant cells, which collectively dictate disease progression [4, 5]. Emerging evidence suggests that RiboSis dysregulation may alter immune cell recruitment and checkpoint expression, impacting response to immunotherapy [6]. However, comprehensive analyses integrating RiboSis‐associated gene networks, TME dynamics, and therapeutic vulnerabilities are lacking.

Herein, we employed an integrative multi‐omics framework coupled with advanced machine learning algorithms [7] to systematically delineate RiboSis‐associated molecular signatures in clear cell renal cell carcinoma (KIRC). Through synergistic analysis of single‐cell RNA sequencing (scRNA‐seq) and bulk transcriptomic data, we identified robust gene modules correlated with RiboSis using high‐dimensional weighted gene co‐expression network analysis (hdWGCNA), followed by the establishment of prognostic clusters via the Partitioning Around Medoids (PAM) algorithm. Subsequent machine learning‐based feature selection and refinement identified PTGER4, a G‐protein‐coupled receptor, as a pivotal tumour suppressor. Comprehensive functional characterisation revealed its immunomodulatory properties, predictive potential for immunotherapy response, and significant associations with therapeutic sensitivity. In vitro functional assays further substantiated the tumour‐suppressive role of PTGER4. This study provides mechanistic insights into the tripartite interplay between RiboSis, immune microenvironment remodelling, and treatment responsiveness in KIRC, establishing a translational paradigm for precision oncology approaches.

## 2 Materials and Methods

### 2.1 Dataset Collection and Processing

The scRNA‐seq dataset was sourced from GSE159115 [8]. The bulk RNA sequencing dataset was collected from TCGA (The Cancer Genome Atlas) [9], GTEx (Genotype‐Tissue Expression) [10], and ICGC (The International Cancer Genome Consortium, RECA‐EU). The KIRC microarray chip datasets included E‐MTAB‐1980 [11] from ArrayExpress.

### 2.2 Computational Analysis

The 331 RiboSis‐related genes were obtained from the previous finding [12]. The R package Seurat [13] was implemented to process the KIRC scRNA‐seq dataset, and Uniform Manifold Approximation and Projection (UMAP) was used to present the distribution of cells. Cancer cells were extracted for the hdWGCNA analysis [14] to determine the most correlated gene modules related to the RIBOSIS feature. A soft power threshold of seven was selected to construct a scale‐free co‐expression network. The hdWGCNA was selected because it is a state‐of‐the‐art method specifically designed to extract robust, functionally coherent gene co‐expression networks from the challenging context of scRNA‐seq data, thereby directly addressing our need to define a RiboSis‐associated transcriptional program within the KIRC ecosystem. The R package ConsensusClusterPlus [15] was used to identify the RiboSis patterns using the PAM method. The PAM algorithm, enhanced by a consensus clustering framework, was chosen for its proven robustness, stability, and ability to yield clearly defined and clinically interpretable patient subtypes from high‐dimensional molecular data, making it ideal for establishing the prognostic RiboSis patterns in KIRC. Differentially expressed genes (DEGs) between RiboSis patterns were identified using the limma R package [16], applying a threshold of absolute log2 fold change (|logFC|) > 1. Kyoto Encyclopedia of Genes and Genomes (KEGG) enrichment analysis was performed on the DEGs [17]. Pattern genes (DEGs between PAM‐based RiboSis patterns) and hdWGCNA genes (yellow module genes) were intersected. Univariate Cox regression analysis was performed to ascertain their prognostic values. CoxBoost [18] and Random Survival Forest [19] were applied for the dimensional reduction of these prognostically important genes.

We collected nine immunotherapy determinants [20], including immune cytolytic activity (CYT) [21], interferon‐gamma immune signature (IFNγIS) [22], expanded immune signature (AyersExpIS) [22], T cell‐inflamed signature (GEP) [22], Roh immune score (RohIS) [23], Davoli immune signature (DavoliIS) [24], repressed immune resistance (RIR) [25], ImmuneScore [26], and network‐based ICB immunotherapeutic signature (ICBnetIS) [20], to assist immunotherapy prediction [27]. The Kaplan–Meier (KM) survival curves for overall survival (OS) were plotted and compared using the R package survminer. The immune infiltration characteristics related to PTGER4 were predicted using the Estimation of Stromal and Immune cells in Malignant Tumour tissues using Expression (ESTIMATE) algorithm [26], Microenvironment Cell Populations‐counter (MCPcounter) [28], Porpimol's study [29], and Tumour Immune Estimation Resource (TIMER) [24, 30]. The cancer immune cycle [31] in KIRC was quantified. The connections between PTGER4 and seven immunomodulator categories were explored [32, 33]. Immunotherapy prediction of PTGER4 was performed using the BEST platform. Gene set enrichment analysis (GSEA) of KEGG terms and the Metascape platform were employed for pathway annotation of PTGER4 [34]. Drug sensitivity to PTGER4 was predicted using oncoPredict [35], which employs a ridge regression model trained on the Genomics of Drug Sensitivity in Cancer (GDSC) database to predict the half‐maximal inhibitory concentration (IC₅₀) of compounds in tumour samples. The mutation characteristics of PTGER4 were analysed via GISTIC 2.0 analysis [36].

### 2.3 Cell Counting Kit‐8 (CCK‐8) Assay

Cells were plated in 96‐well plates at a seeding density of 1000 cells per well. The cells were treated with siPTGER4 for 24 h. Subsequently, the CCK‐8 reagent was used, and the optical density at 450 nm was measured.

### 2.4 EDU Assay

Cells were seeded in 6‐well plates and subjected to siRNA treatment for 24 h. Subsequently, the cells were incubated with EDU (Beyotime, China) for 2 h and DAPI for 10 min. Fluorescence imaging was then carried out to visualise EdU‐positive cells (stained green) and nuclei counterstained with DAPI (stained blue). The EdU incorporation rate was determined by calculating the ratio of the number of EdU‐positive cells to the number of DAPI‐positive cells. To ensure the robustness and reliability of the experimental results, data analysis was based on a minimum of three independent biological replicates.

### 2.5 Conoly Formation Assay

Cells were seeded in 6‐well plates at a low density of 500 cells per well and subjected to siRNA treatment for 24 h. Subsequently, the culture medium was replaced with fresh complete medium, and the cells were incubated for 10–14 days to allow for colony formation, with the medium being refreshed every 3 days. Following the incubation period, the cells were washed with phosphate‐buffered saline (PBS), fixed with 4% paraformaldehyde for 15 min, and then stained with a 0.1% crystal violet solution for 30 min at room temperature. After extensive washing and air‐drying, macroscopic colonies were manually counted. The colony formation rate was determined by calculating the ratio of the number of colonies to the number of seeded cells. To ensure the robustness and reliability of the experimental results, data analysis was based on a minimum of three independent biological replicates.

### 2.6 RNA Extraction and Real‐Time PCR

Cells were rinsed with phosphate‐buffered saline (PBS) and lysed using the RNA Isolator Total RNA Extraction Reagent (Vazyme, China). Following this, 1 μg of messenger RNA (mRNA) was reverse‐transcribed into complementary DNA (cDNA) with the use of HiScript II Q RT SuperMix for quantitative polymerase chain reaction (qPCR; Vazyme, China). The sequences of the primers are presented in Table 1. Taq Pro Universal SYBR qPCR Master Mix (Vazyme, Q712‐02) was utilised to amplify the cDNA. The fluorescence generated during the amplification process was measured by means of a real‐time PCR detection system (Bio‐Rad, USA). The relative expression level of the target mRNA was calculated by applying the 2^−ΔΔCt^ method.

**TABLE 1:** Primer sequences for RT‐PCR.

Gene	Primer sequence (5′–3′)
*ACTB*	
F	TCCTTCCTGGGCATGGAGTC
R	CTTCTGCATCCTGTCGGCAAT
*PTGER4*	
F	CCGGCGGTGATGTTCATCTT
R	CCCACATACCAGCGTGTAGAA
*MMP2*	
F	TACAGGATCATTGGCTACACACC
R	GGTCACATCGCTCCAGACT
*MMP9*	
F	TGTACCGCTATGGTTACACTCG
R	GGCAGGGACAGTTGCTTCT
*PCNA*	
F	CCTGCTGGGATATTAGCTCCA
R	CAGCGGTAGGTGTCGAAGC
*KI67*	
F	ACGCCTGGTTACTATCAAAAGG
R	CAGACCCATTTACTTGTGTTGGA

## 3 Results

### 3.1 Identification of RiboSis‐Related Gene Module

**FIGURE 1:**
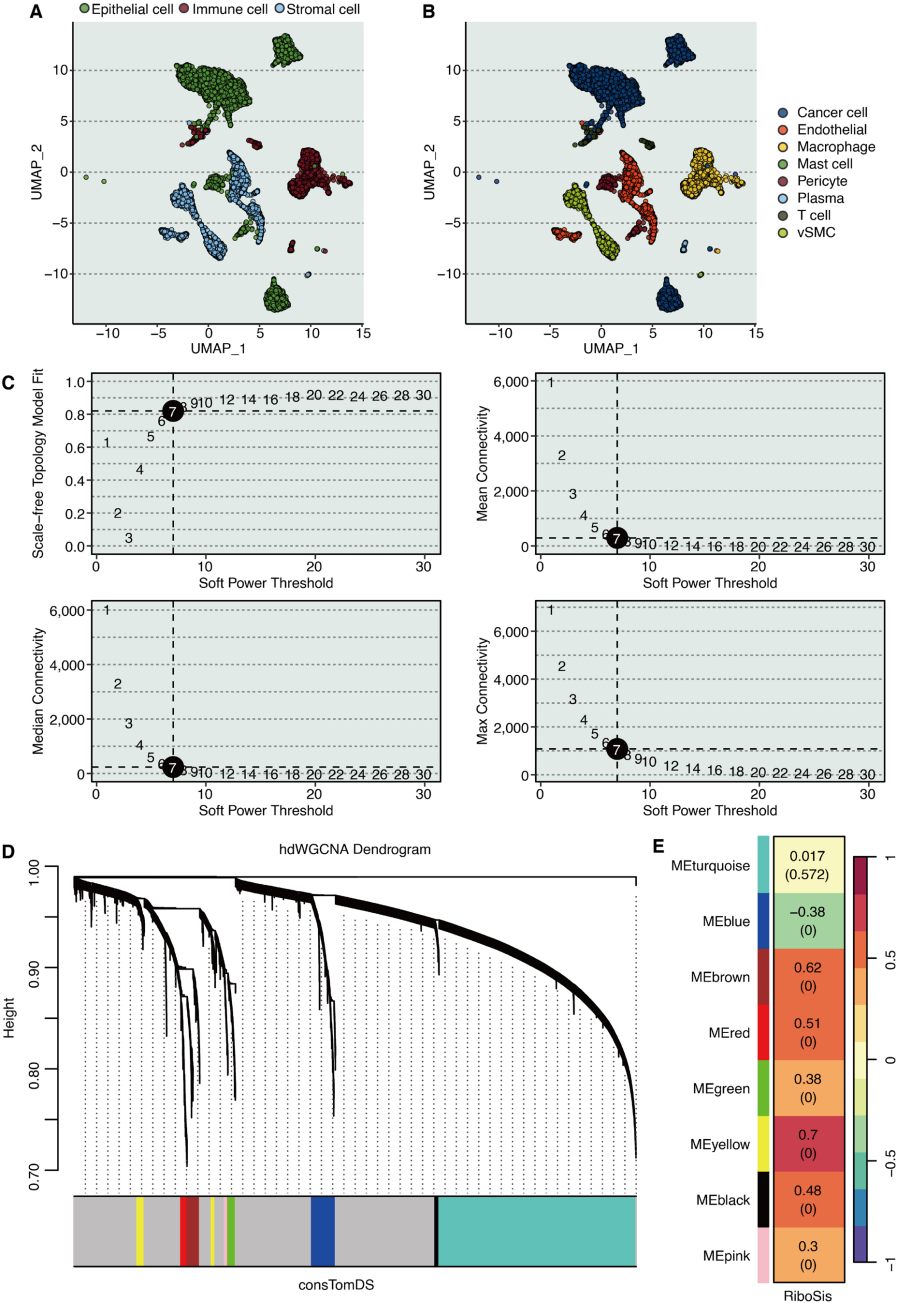
Identification of RiboSis‐associated gene modules in KIRC. (A) UMAP projection illustrating major cellular compartments within the KIRC tumour microenvironment. (B) UMAP delineation of rare cell populations in the KIRC tumour microenvironment. (C) Optimization of soft‐thresholding power in hdWGCNA, assessing scale‐free topology fit, mean, median, and maximum connectivity. (D) Waterfall plot depicting gene module distribution via hdWGCNA. (E) Correlation analysis between the RiboSis signature and identified gene modules.

The TME of KIRC contains three major cell populations: immune cells, epithelial cells, and stromal cells (Figure 1A). Further analysis revealed additional cell types, including endothelial cells, macrophages, mast cells, pericytes, plasma cells, vascular smooth muscle cells (vSMC), and T cells (Figure 1B). The hdWGCNA algorithm identified eight as the optimal soft‐threshold power (Figure 1C). Gene modules derived from hdWGCNA are presented in Figure 1D, with the yellow module showing the strongest association with the RiboSis feature (Figure 1E).

### 3.2 Development of RiboSis Patterns

**FIGURE 2:**
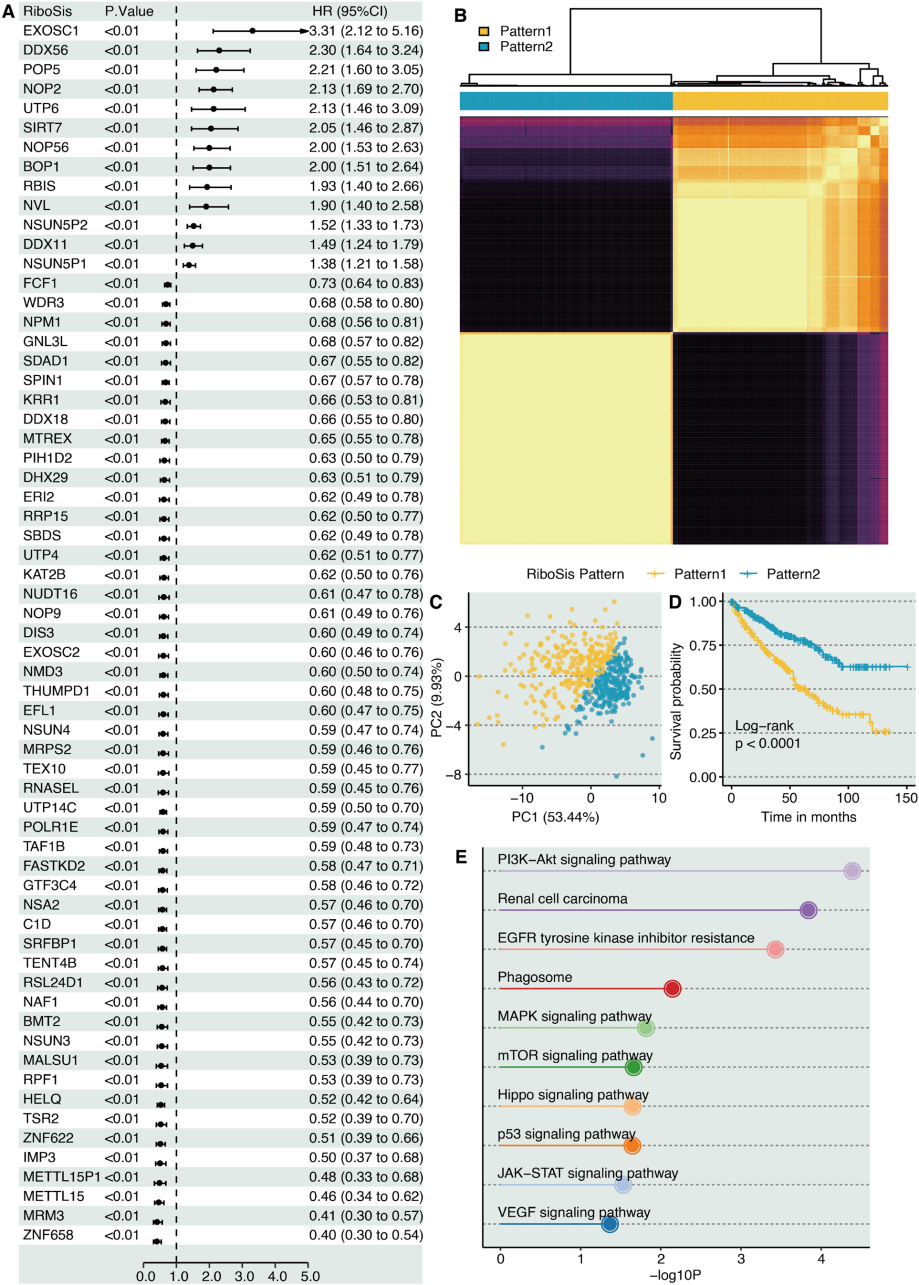
Characterisation of RiboSis molecular subtypes in KIRC. (A) Univariate Cox regression assessing prognostic significance of RiboSis‐related genes. (B) Heatmap representing PAM‐derived RiboSis subtypes. (C) Principal component analysis (PCA) of KIRC samples stratified by RiboSis subtypes. (D) Kaplan–Meier survival analysis across distinct RiboSis subtypes. (E) KEGG pathway enrichment of differentially expressed genes (DEGs) between RiboSis subtypes.

Univariate Cox regression analysis was performed on RiboSis‐related genes and identified 93 prognostic genes (Figure 2A). Using PAM clustering, we identified two distinct RiboSis patterns (Figure 2B). KIRC samples clearly segregated into these two subgroups via principal component analysis (PCA) (Figure 2C). Notably, patients of RiboSis pattern 1 exhibited significantly longer OS (Log‐rank, *p* < 0.0001) compared to pattern 2 (Figure 2D). KEGG enrichment analysis on DEGs between the RiboSis patterns revealed that oncogenic pathways including MAPK signalling pathway, Hippo signalling pathway, and mTOR signalling pathway were enriched (Figure 2E).

### 3.3 Identification of PTGER4 as a Critical Tumour Suppressor

**FIGURE 3:**
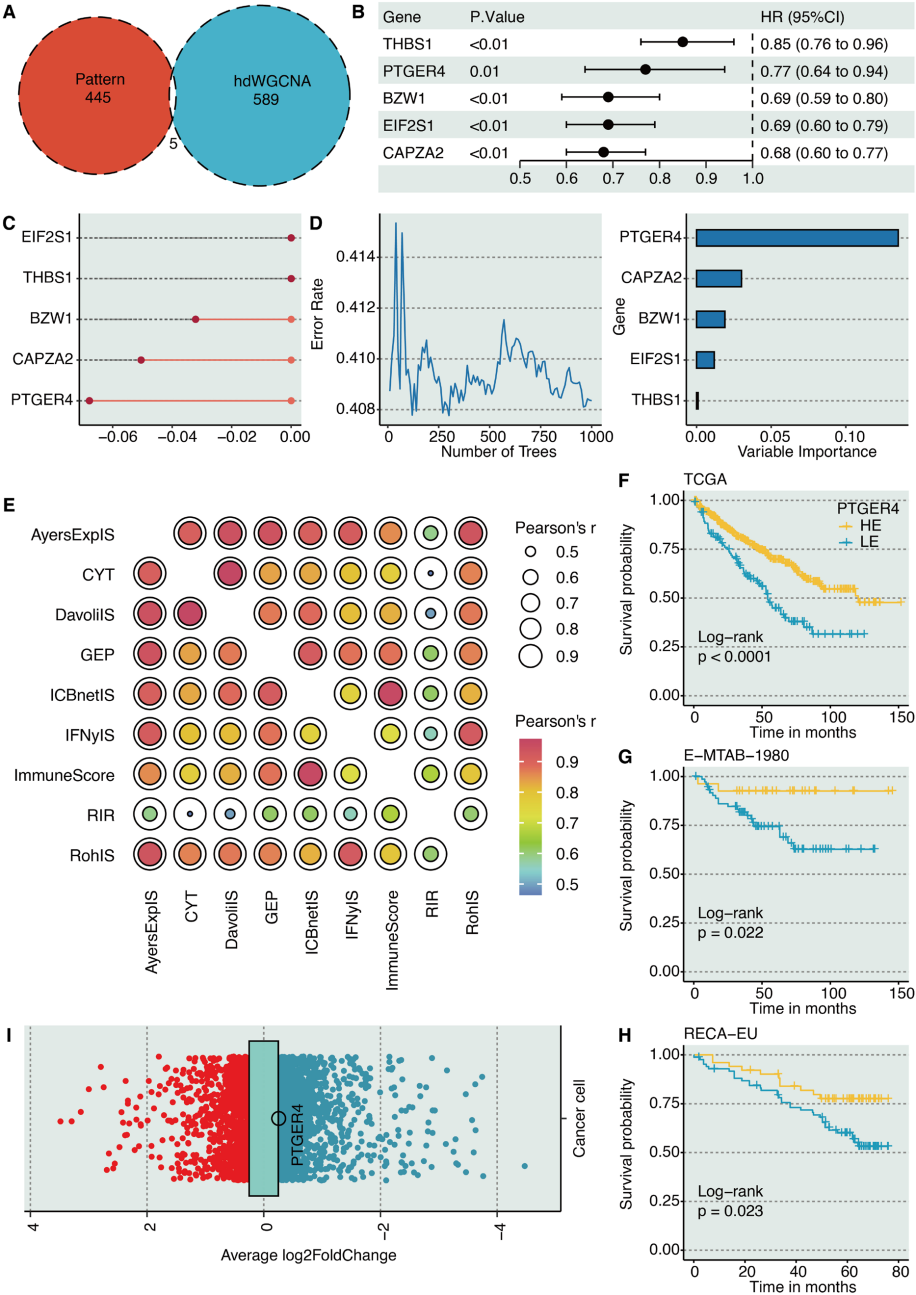
PTGER4 as a prognostic and tumour‐suppressive hub in KIRC. (A) Venn diagram intersecting DEGs from RiboSis subtypes and hdWGCNA‐derived yellow module genes. (B) Univariate Cox regression evaluating survival‐associated intersected genes. (C, D) Feature selection via CoxBoost and Random Survival Forest algorithms for prognostic gene prioritisation. (E) Correlation network of nine immunotherapy response determinants. (F–H) Kaplan–Meier survival analysis of PTGER4‐stratified cohorts (TCGA‐KIRC, E‐MTAB‐1980, RECA‐EU). (I) Volcano plot demonstrating PTGER4 downregulation in malignant versus non‐malignant cells at single‐cell resolution.

The five intersected genes were found between RiboSis pattern‐related genes (DEGs between two RiboSis patterns) and hdWGCNA genes (yellow module genes) (Figure 3A). Univariate Cox regression analysis revealed five prognostically important genes (Figure 3B). Two ML approaches were employed to refine the prognostic gene set from the initial five candidates: CoxBoost, a semi‐parametric model‐based boosting algorithm, identified three key genes (including PTGER4) that provided the most parsimonious model for predicting overall survival (Figure 3C). This method effectively penalises non‐informative features, reducing the risk of overfitting. Random Survival Forest (RSF), a non‐parametric tree‐based ensemble method, was used in parallel. It ranked all five genes by their variable importance, a measure of how much each gene contributes to improving prediction accuracy across thousands of decision trees (Figure 3D). These dimension reduction techniques collectively facilitated the identification of core prognostic biomarkers while maintaining model robustness. As a result, PTGER4 was found to be the most potent biomarker. Surprisingly, KIRC samples with high PTGER4 expression had significantly prolonged survival time in the TCGA‐KIRC cohort (Figure 3F), E‐MTAB‐1980 cohort (Figure 3G), and RECA‐EU cohort (Figure 3H). Besides, PTGER4 was significantly downregulated in cancer cells (logFC = −0.256) compared to non‐cancerous cells at the scRNA‐seq level (Figure 3I).

### 3.4 Immunotherapy Prediction of PTGER4

**FIGURE 4:**
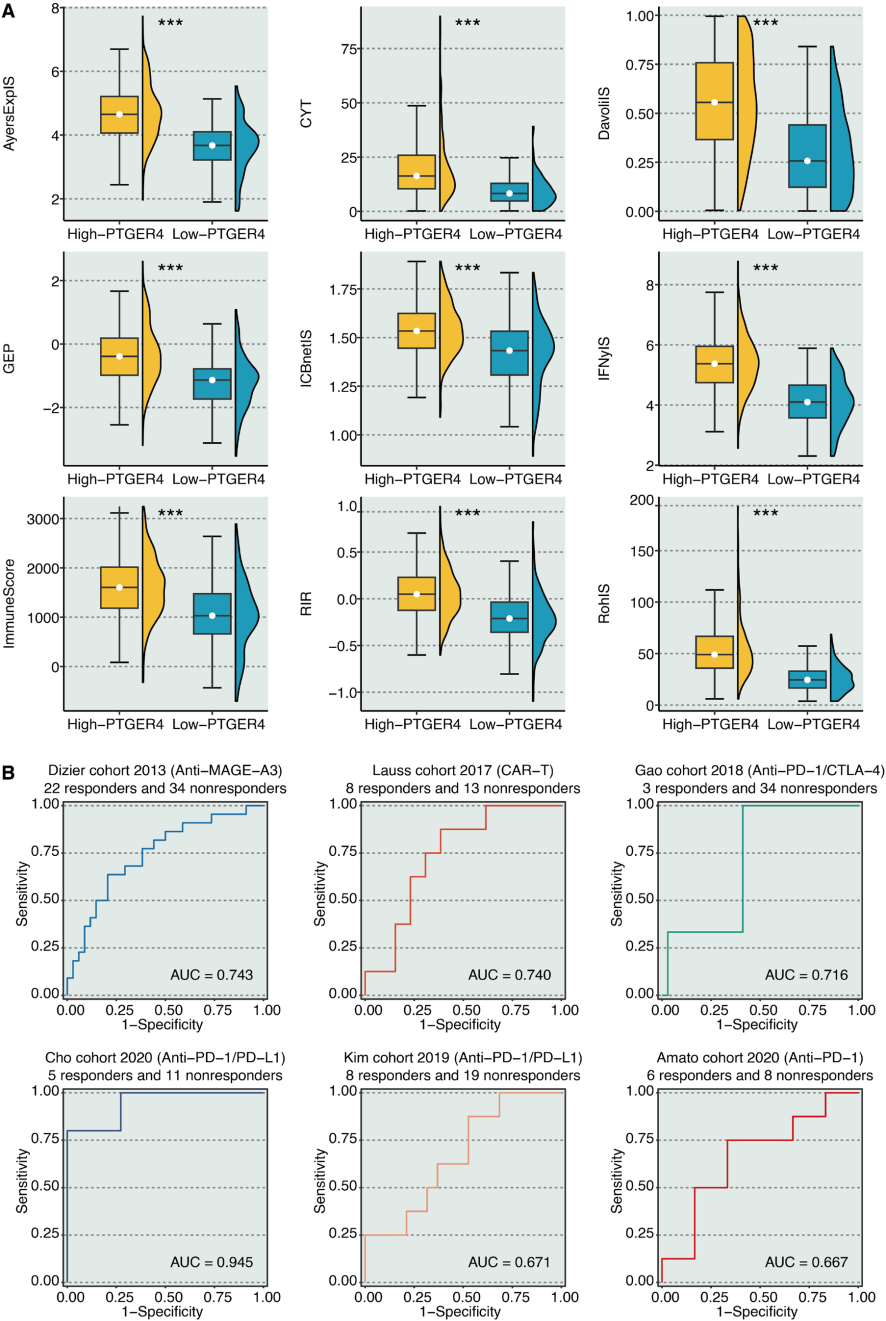
Predictive value of PTGER4 in immunotherapy response. (A) Comparative analysis of immunotherapy determinants across PTGER4‐defined subgroups. (B) Receiver operating characteristic (ROC) curves evaluating PTGER4's predictive performance in six independent cohorts. ***p < 0.001.

The levels of nine immunotherapy determinants, including CYT, IFNγIS, AyersExplS, GEP, RohIS, DavoliIS, RIR, ImmuneScore, and ICBnetIS, were estimated. The intercorrelation between the nine immunotherapy determinants was robust, indicating their reliability (Figure 3E). Meanwhile, these determinants were all significantly higher in KIRC samples with high PTGER4 expression (Figure 4A). In addition, the AUC values of PTGER4 in predicting immunotherapy responses were larger than 0.6 in six cohorts, including Dizier cohort 2013 (Anti‐MAGE‐A3), Lauss cohort 2017 (CAR‐T), Gao cohort 2018 (Anti‐PD‐1/CTLA‐4), Cho cohort 2020 (Anti‐PD‐1/PD‐L1), Kim cohort 2019 (Anti‐PD‐1/PD‐L1), and Amato cohort 2020 (Anti‐PD‐1) (Figure 4B).

### 3.5 Immune Features of PTGER4

**FIGURE 5:**
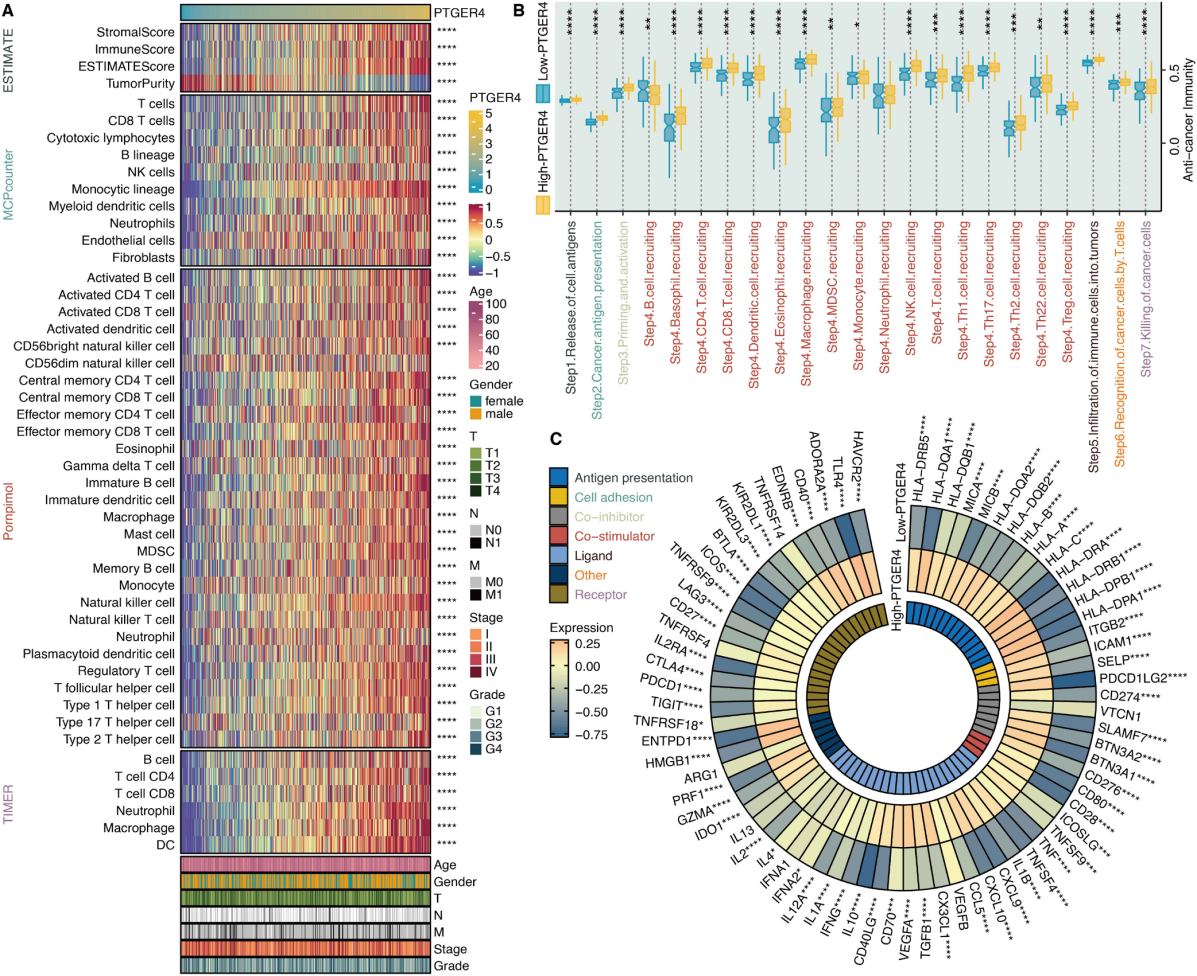
Immunomodulatory role of PTGER4 in KIRC. (A) Correlation heatmaps linking PTGER4 expression with tumour microenvironment scores and immune infiltrates (TIMER, Pornpimol, MCPcounter). (B) Differential activity of cancer‐immunity cycle steps between PTGER4‐high and PTGER4‐low groups. (C) Association between PTGER4 and immune checkpoint modulators. *p < 0.05, **p < 0.01, ***p < 0.001, ****p < 0.0001.

PTGER4 had a significantly positive correlation with microenvironment scores (stromal, immune, estimate), TIMER‐based immune cells (B cells, CD4 T cells, CD8 T cells, neutrophils, macrophages, dendritic cells), Pornpimol‐based immune cells (macrophages, B cells, T cells, natural killer cells), and MCPcounter‐based immune cells (T cells, B cells, neutrophils) (Figure 5A). Most steps of the cancer immune cycle were highly activated in KIRC samples with high PTGER4 expression (Figure 5B). Besides, PTGER4 was significantly negatively associated with immune modulators, such as CD40, PDCD1, CTLA4, TIGIT, CD276, CD80, BTLA, HAVCR2, and CD274 (Figure 5C).

### 3.6 Functional Annotation of PTGER4

**FIGURE 6:**
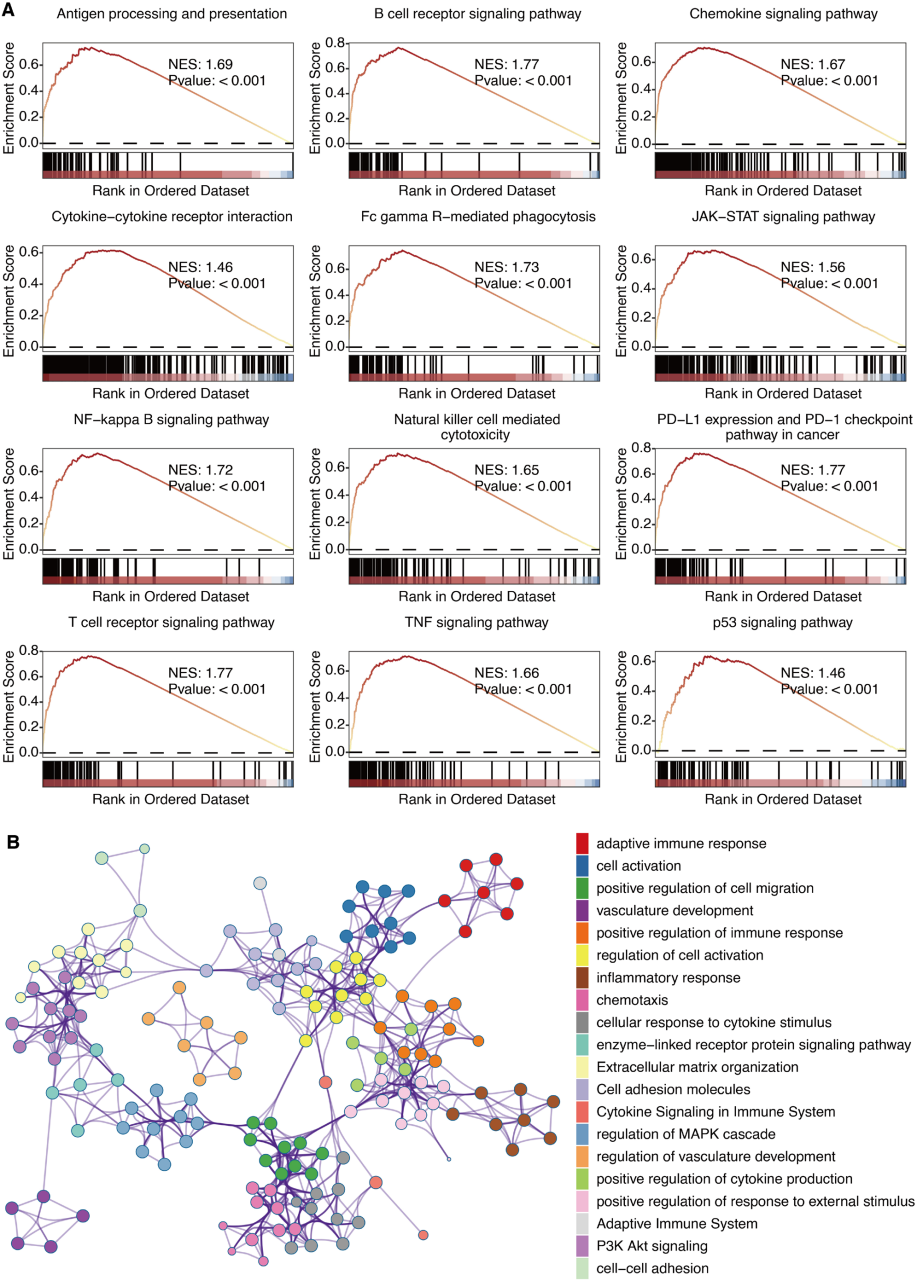
Functional landscape of PTGER4 in KIRC. (A) Gene Set Enrichment Analysis (GSEA) of KEGG pathways associated with PTGER4 expression. (B) Metascape‐derived pathway enrichment of PTGER4‐coexpressed genes.

KEGG pathways, including B cell activity, T cell activity, natural killer cell cytotoxicity, inflammatory activity, and immune response, were positively related to PTGER4 (Figure 6A). Metascape‐based pathways, including immune activity and inflammatory activity, were also positively associated with PTGER4 (Figure 6B).

### 3.7 Drug Prediction of PTGER4

**FIGURE 7:**
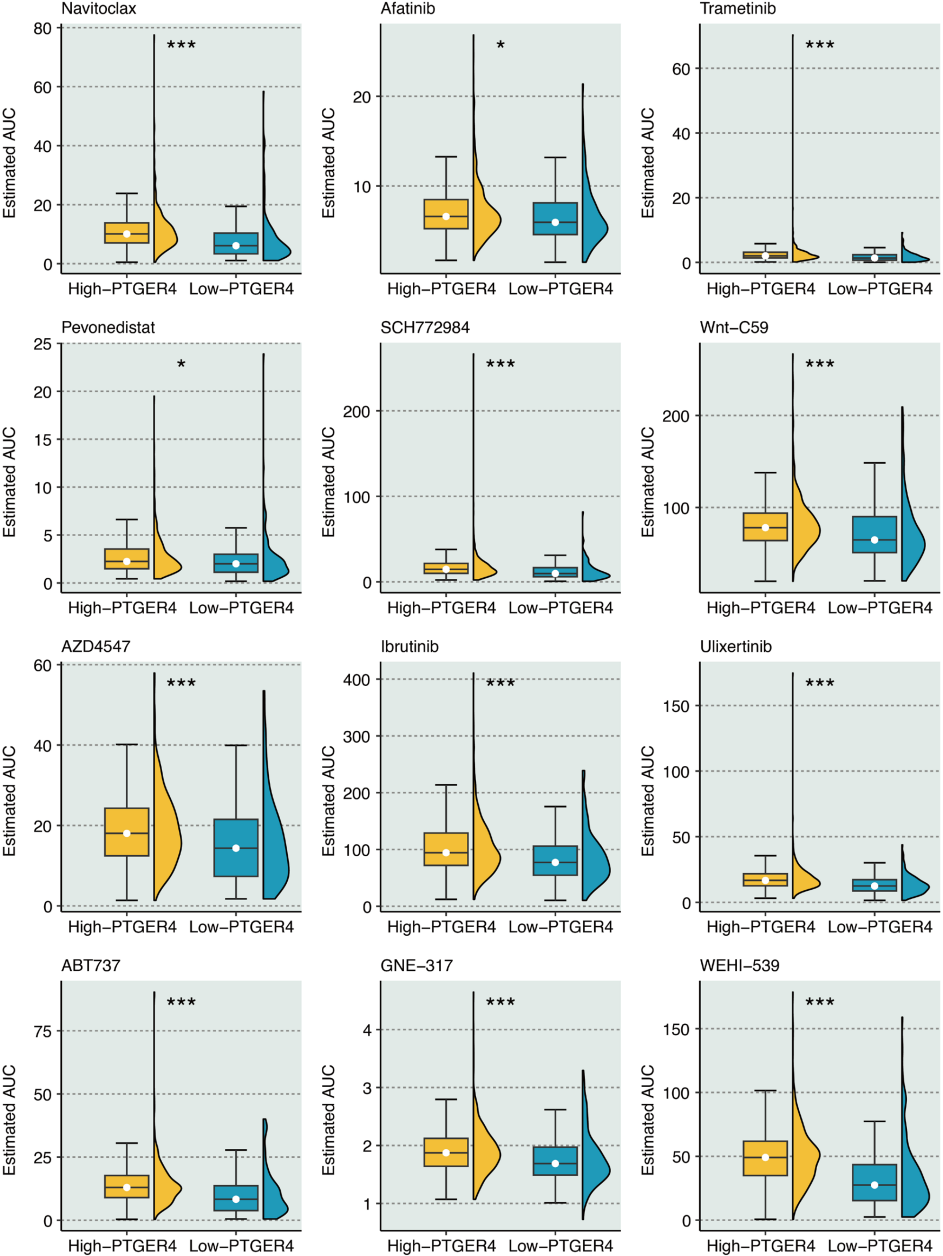
Therapeutic vulnerabilities associated with PTGER4 status. Differential drug sensitivity (AUC estimates) to 12 compounds in PTGER4‐stratified KIRC models. *p < 0.05, ***p < 0.001.

12 drugs, including Navitoclax, Afatinib, Trametinib, Pevonedistat, SCH772984, Wnt‐C59, AZD4547, Ibrutinib, Ulixertinib, ABT737, GNE‐317, and WEHI‐539, had significantly higher responses in KIRC samples with high PTGER4 expression (Figure 7).

### 3.8 Mutation Characteristics of PTGER4

**FIGURE 8:**
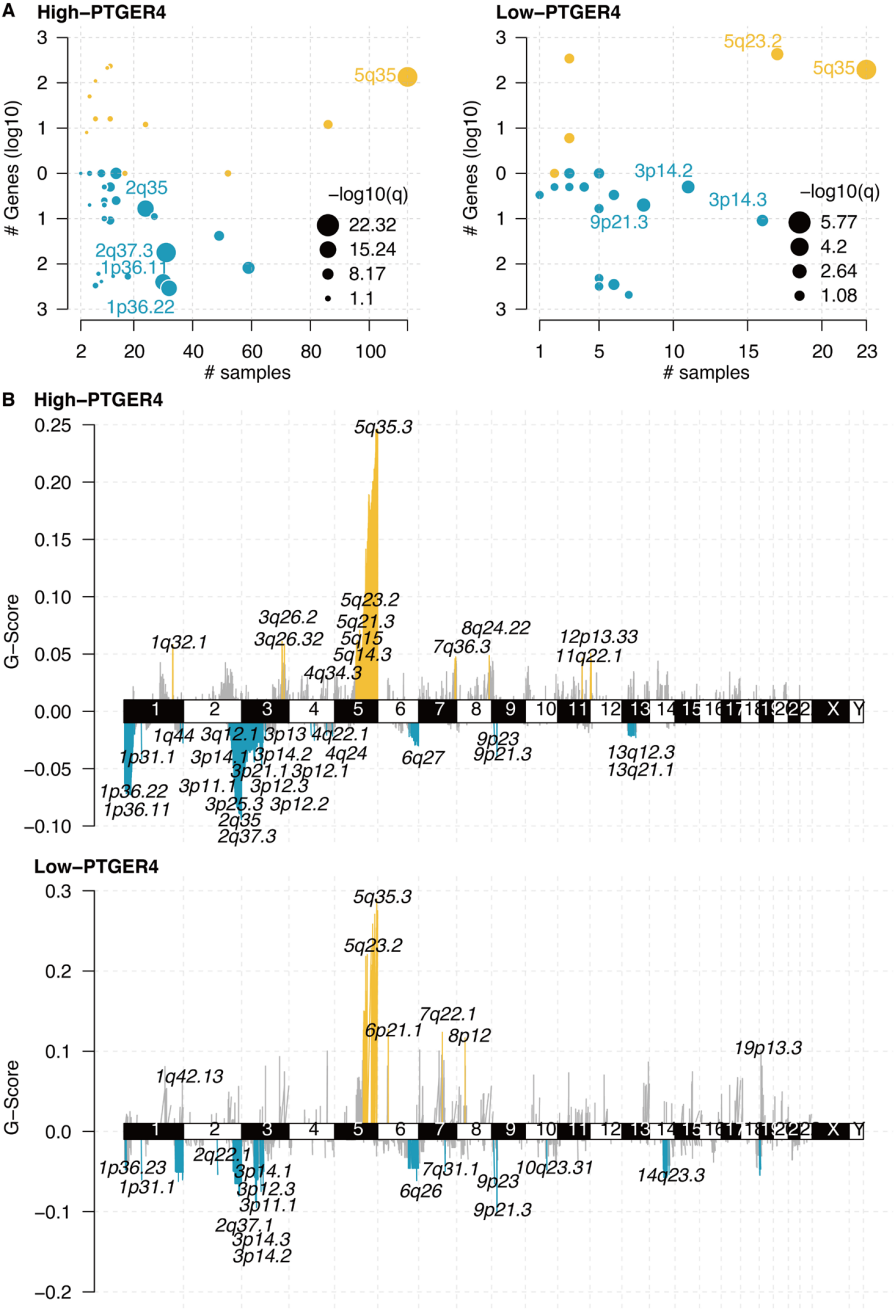
Genomic alterations linked to PTGER4 expression. (A) Oncoprint of somatic mutations enriched in PTGER4‐defined subgroups. (B) G‐scores quantifying mutation burdens across PTGER4‐based groups.

The mutated genes in the PTGER4‐based groups are shown in Figure 8A,B, where 2q35, 2q37.3, 1p36.11, and 1p36.22 were highly mutated in KIRC samples with high PTGER4 expression. The 1p36 locus is a well‐characterised tumour suppressor hub frequently deleted in diverse cancers, including KIRC [37]. This region contains several genes implicated in chromatin remodelling (e.g., CHD5), apoptosis, and cell cycle regulation. The co‐occurrence of high PTGER4 expression and 1p36 deletion suggests a potential compensatory relationship, where the loss of tumour suppressors at 1p36 may be counterbalanced by the upregulation of PTGER4‐mediated tumour‐suppressive pathways, such as immune activation.

### 3.9 In Vitro Validation on PTGER4

**FIGURE 9:**
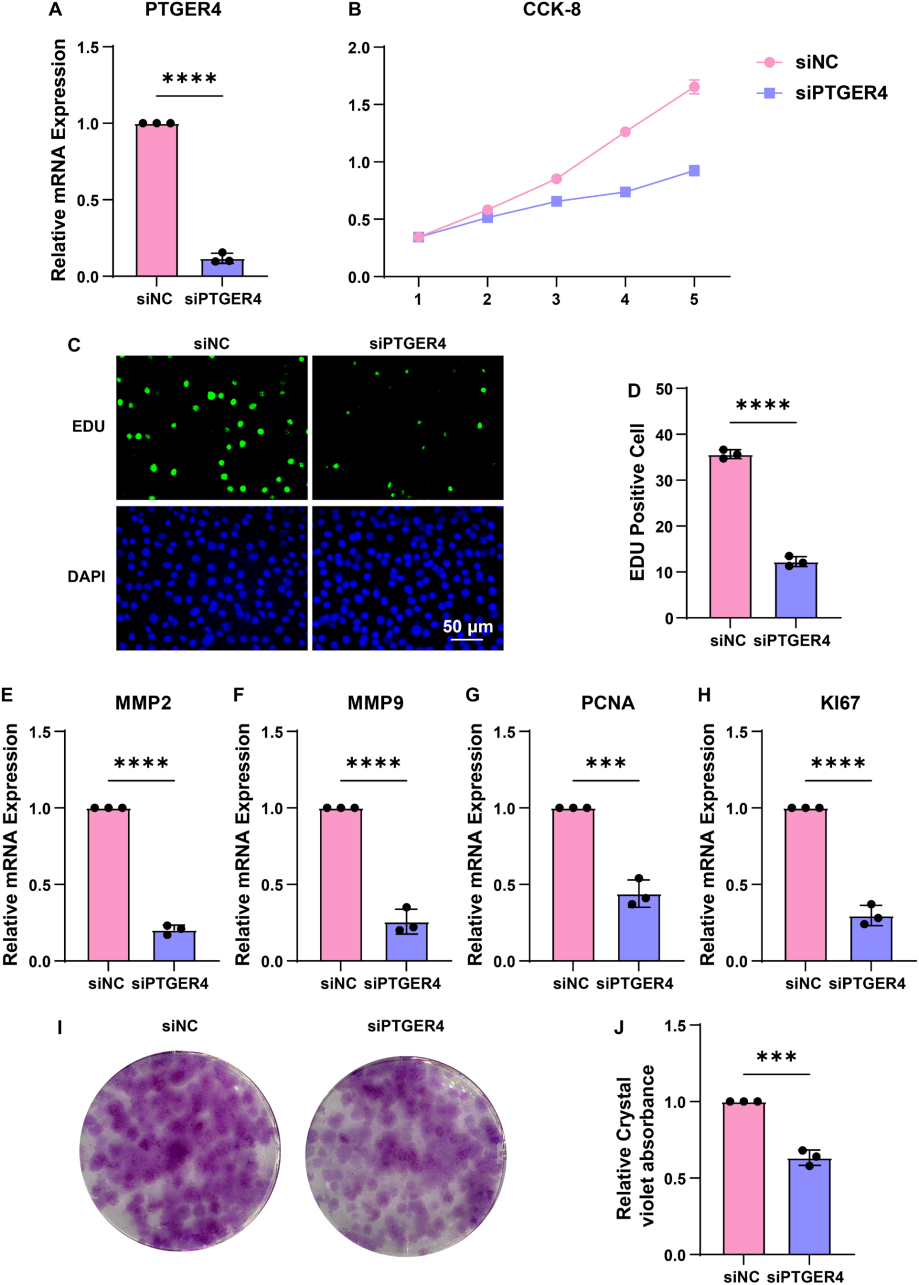
Experimental validation of PTGER4's tumour‐suppressive function. (A) qRT‐PCR confirming PTGER4 knockdown efficiency in KIRC cell lines. (B) CCK‐8 assays quantifying proliferative capacity post‐PTGER4 silencing. (C, D) EdU incorporation assays (imaging and quantification) assessing DNA replication activity. (E–H) qRT‐PCR analysis of MMP2, MMP9, PCNA, and KI67 expression following PTGER4 depletion. (I, J) Colony formation assays (representative images and absorbance quantification) post‐PTGER4 knockdown. ***p < 0.001, ****p < 0.0001.

We further performed in vitro validation on PTGER4. The mRNA expression of PTGER4 was significantly inhibited after siRNA transfection (Figure 9A). The OD values of the tumour cells were significantly reduced in the siRNA group (Figure 9B). Besides, the positive EdU‐stained tumour cells were significantly reduced in the siRNA group (Figure 9C,D). Classical tumour markers, including MMP2 (Figure 9E), MMP9 (Figure 9F), PCNA (Figure 9G), and Ki67 (Figure 9H), had significantly decreased mRNA expression in the siRNA group. Moreover, the colony formation of tumour cells was significantly reduced in the siRNA group (Figure 9I,J).

### 3.10 Pan‐Cancer Role of PTGER4

**FIGURE 10:**
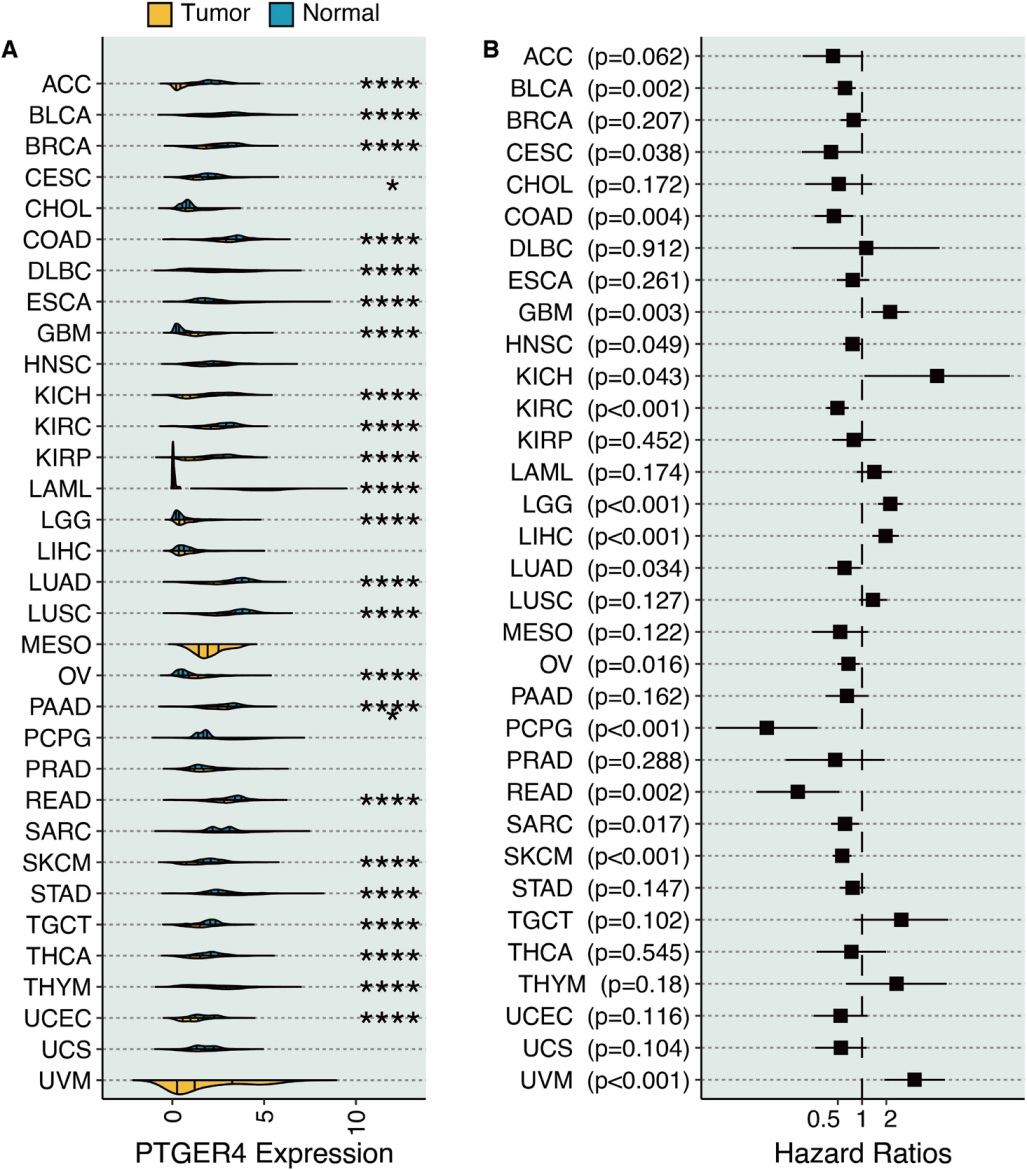
Pan‐cancer role of PTGER4. (A) Violin plot illustrates PTGER4 expression differences between tumour and adjacent normal tissues. (B) Hazard ratios derived from univariate Cox regression indicate PTGER4's prognostic value. *p < 0.05, ****p < 0.0001.

PTGER4 expression was significantly lower in tumour and normal tissues in most cancer types (Figure 10A). Univariate Cox regression analysis of PTGER4 confirmed that PTGER4 was a protective marker in most cancer types (Figure 10B).

## 4 Discussion

Herein, we systematically delineate the prognostic and immunotherapeutic relevance of RiboSis‐associated molecular signatures in clear cell renal cell carcinoma (KIRC). Through integrative multi‐omics analysis combining single‐cell and bulk transcriptomic profiling, we identified two clinically distinct RiboSis subtypes exhibiting significantly divergent survival outcomes. The favourable‐prognosis subgroup (Pattern 1) demonstrated significant enrichment of established oncogenic pathways, including MAPK and mTOR signalling cascades, consistent with the emerging paradigm of RiboSis‐mediated metabolic reprogramming in tumour progression. Leveraging high‐dimensional WGCNA and intersectional bioinformatics approaches, we identified PTGER4 as a master regulatory node within the RiboSis‐associated gene network. Our ML‐driven workflow was not merely a computational exercise; it was a necessary strategy to distill robust biological signals and clinically actionable biomarkers from the intricate interplay of genes, cells, and pathways that define KIRC heterogeneity, a task for which conventional analysis pipelines are often underpowered or ill‐suited [38, 39, 40]. Subsequent mechanistic characterisation revealed its pivotal role in modulating KIRC pathogenesis, positioning it as both a prognostic biomarker and potential therapeutic target.

PTGER4 emerged as a potent tumour suppressor [41], with high expression correlating with improved survival across independent cohorts. PTGER4 SNPs might affect the susceptibility to gastric cancer [42]. Its downregulation in cancer cells at the scRNA‐seq level suggests a role in malignant transformation. Mechanistically, PTGER4's association with enhanced immune infiltration (e.g., CD8+ T cells, dendritic cells) and elevated immunotherapy determinants (e.g., CYT, IFNγIS) [29] implies its capacity to remodel the TME into an immunoreactive state. This aligns with findings by Vésteinn Thorsson et al., who identified immune gene signatures as predictors of checkpoint blockade response [32]. Furthermore, PTGER4's negative correlation with immune checkpoints (PDCD1, CTLA4) underscores its potential to counteract immune exhaustion, a phenomenon critical for sustaining anti‐tumour immunity.

The robust predictive performance of PTGER4 for immunotherapy response (AUC > 0.6 in six cohorts) highlights its clinical utility. Its association with activated cancer immune cycle steps, such as antigen presentation and T cell recruitment, further supports its role in fostering an immune‐permissive TME. These findings resonate with the framework proposed by Chen and Mellman, wherein immune cycle activation dictates therapeutic efficacy [31]. Functional annotation revealed PTGER4's involvement in B cell activity, NK cell cytotoxicity, and inflammatory responses, pathways previously linked to immune‐mediated tumour control. Additionally, PTGER4‐high tumours exhibited sensitivity to 12 targeted therapies, including MEK inhibitors (Trametinib) and BTK inhibitors (Ibrutinib), suggesting combinatorial therapeutic strategies. For instance, coupling PTGER4‐enhancing agents with immune checkpoint inhibitors could synergistically augment anti‐tumour immunity.

The CCK‐8 assay revealed a significant reduction in optical density (OD) values upon PTGER4 silencing (*p* < 0.05), corroborating its essential role in sustaining tumour cell proliferation. These findings are consistent with PTGER4's tumour‐suppressive function, wherein its downregulation attenuates oncogenic growth kinetics. Furthermore, EdU incorporation assays demonstrated a pronounced decrease in proliferating tumour cells in the siRNA‐treated group (*p* < 0.01), reinforcing PTGER4's regulatory influence on cell cycle progression and mitotic activity. Quantitative RT‐PCR analysis revealed a concomitant downregulation of established tumorigenic markers, including MMP2, MMP9, PCNA, and Ki67 (*p* < 0.05), following PTGER4 knockdown. Given the well‐characterised roles of MMP2 and MMP9 in extracellular matrix degradation and metastatic dissemination, their suppression suggests a mechanistic link between PTGER4 deficiency and impaired tumour invasiveness. Additionally, the marked reduction in PCNA and Ki67 expression further underscores PTGER4's regulatory impact on proliferative signalling cascades. Supporting these observations, Transwell migration assays exhibited a significant decline in tumour cell motility upon PTGER4 silencing (*p* < 0.01), indicative of its role in modulating metastatic potential. Collectively, these findings establish PTGER4 as a pivotal tumour suppressor in KIRC, governing both proliferative capacity and invasive behaviour through the regulation of key oncogenic effectors.

Despite these advances, limitations exist. The retrospective nature of public cohorts necessitates prospective validation of PTGER4's predictive power. Experimental studies are required to delineate PTGER4's mechanistic role in RiboSis regulation and immune modulation. Moreover, the lack of proteomic data limits insight into post‐transcriptional regulation of PTGER4 [43].

In conclusion, our findings identify PTGER4 as a pivotal biomarker in KIRC, integrating RiboSis dysregulation, immune modulation, and therapeutic responsiveness. However, the current in vitro validation would benefit from including plans for future in vivo studies or co‐culture models to validate immune interactions. Further investigations should focus on developing PTGER4‐targeted therapeutic strategies and rigorously validating their clinical efficacy through prospective trials, thereby facilitating the advancement of precision medicine in KIRC management.

## Author Contributions

Jian Huang designed and supervised the study. Hanjing Zhou conducted the analyses and wrote the initial manuscript draft. Zirui Li, Jun Ying, Yan Liu, and Xuchun Xu critically reviewed and revised the manuscript. All authors have read and approved the final version of the manuscript.

## Funding

This research is affiliated with the project fund, a key science and technology project in Jinhua City (2021‐3‐073).

## Ethics Statement

The authors have nothing to report.

## Consent

The authors have nothing to report.

## Conflicts of Interest

The authors declare no conflicts of interest.

## Data Availability

The datasets generated and analyzed during this study are available from the corresponding author upon reasonable request.

## References

[1] J. J. Hsieh , M. P. Purdue , S. Signoretti , et al., “Renal Cell Carcinoma,” Nature Reviews. Disease Primers 3 (2017): 17009, 10.1038/nrdp.2017.9. PMC5936048 28276433

[2] S. O. Sulima , K. R. Kampen , S. Vereecke , et al., “Ribosomal Lesions Promote Oncogenic Mutagenesis,” Cancer Research 79 (2019): 320–327, 10.1158/0008-5472.CAN-18-1987. 30482776 PMC7116100

[3] J. Pelletier , G. Thomas , and S. Volarevic , “Ribosome Biogenesis in Cancer: New Players and Therapeutic Avenues,” Nature Reviews. Cancer 18 (2018): 51–63, 10.1038/nrc.2017.104. 29192214

[4] S. Chevrier , J. H. Levine , V. R. Zanotelli , et al., “An Immune Atlas of Clear Cell Renal Cell Carcinoma,” Cell 169 (2017): 736–749.e718, 10.1016/j.cell.2017.04.016. 28475899 PMC5422211

[5] Z. Gao , A. Jiang , Z. Li , et al., “Heterogeneity of Intratumoral Microbiota Within the Tumor Microenvironment and Relationship to Tumor Development,” Med Research 1 (2025): 32–61, 10.1002/mdr2.70006.

[6] Z. Song , Y. Wang , M. Zhu , et al., “Exploring Ribosome Biogenesis in Lung Adenocarcinoma to Advance Prognostic Methods and Immunotherapy Strategies,” Journal of Translational Medicine 23 (2025): 503, 10.1186/s12967-025-06489-0. 40316986 PMC12048935

[7] H. Zhang , G. Zhang , P. Xu , et al., “Optimized Dynamic Network Biomarker Deciphers a High‐Resolution Heterogeneity Within Thyroid Cancer Molecular Subtypes,” Med Research 1 (2025): 10–31, 10.1002/mdr2.70004.

[8] Y. Zhang , S. P. Narayanan , R. Mannan , et al., “Single‐Cell Analyses of Renal Cell Cancers Reveal Insights Into Tumor Microenvironment, Cell of Origin, and Therapy Response,” Proceedings of the National Academy of Sciences of the United States of America 118 (2021): e2103240118, 10.1073/pnas.2103240118. 34099557 PMC8214680

[9] D. A. Barbie , P. Tamayo , J. S. Boehm , et al., “Systematic RNA Interference Reveals That Oncogenic KRAS‐Driven Cancers Require TBK1,” Nature 462 (2009): 108–112, 10.1038/nature08460. 19847166 PMC2783335

[10] J. Lonsdale , J. Thomas , M. Salvatore , et al., “The Genotype‐Tissue Expression (GTEx) Project,” Nature Genetics 45 (2013): 580–585, 10.1038/ng.2653. 23715323 PMC4010069

[11] Y. Sato , T. Yoshizato , Y. Shiraishi , et al., “Integrated Molecular Analysis of Clear‐Cell Renal Cell Carcinoma,” Nature Genetics 45 (2013): 860–867, 10.1038/ng.2699. 23797736

[12] Y. Zang , X. Ran , J. Yuan , et al., “Genomic Hallmarks and Therapeutic Targets of Ribosome Biogenesis in Cancer,” Briefings in Bioinformatics 25 (2024): bbae023, 10.1093/bib/bbae023. 38343327 PMC10859687

[13] R. Satija , J. A. Farrell , D. Gennert , A. F. Schier , and A. Regev , “Spatial Reconstruction of Single‐Cell Gene Expression Data,” Nature Biotechnology 33 (2015): 495–502, 10.1038/nbt.3192. PMC4430369 25867923

[14] S. Morabito , F. Reese , N. Rahimzadeh , E. Miyoshi , and V. Swarup , “hdWGCNA Identifies Co‐Expression Networks in High‐Dimensional Transcriptomics Data,” Cell Reports Methods 3 (2023): 100498, 10.1016/j.crmeth.2023.100498. 37426759 PMC10326379

[15] M. D. Wilkerson and D. N. Hayes , “ConsensusClusterPlus: A Class Discovery Tool With Confidence Assessments and Item Tracking,” Bioinformatics 26 (2010): 1572–1573, 10.1093/bioinformatics/btq170. 20427518 PMC2881355

[16] M. E. Ritchie , B. Phipson , D. Wu , et al., “Limma Powers Differential Expression Analyses for RNA‐Sequencing and Microarray Studies,” Nucleic Acids Research 43 (2015): e47, 10.1093/nar/gkv007. 25605792 PMC4402510

[17] M. Kanehisa and S. Goto , “KEGG: Kyoto Encyclopedia of Genes and Genomes,” Nucleic Acids Research 28 (2000): 27–30, 10.1093/nar/28.1.27. 10592173 PMC102409

[18] R. De Bin , “Boosting in Cox Regression: A Comparison Between the Likelihood‐Based and the Model‐Based Approaches With Focus on the R‐Packages CoxBoost and Mboost,” Computational Statistics 31 (2016): 513–531, 10.1007/s00180-015-0642-2.

[19] H. Ishwaran , U. B. Kogalur , E. H. Blackstone , and M. S. Lauer , “Random Survival Forests,” Annals of Applied Statistics 2 (2008): 841–860.

[20] N. Zhang , M. Yang , J. M. Yang , C. Y. Zhang , and A. Y. Guo , “A Predictive Network‐Based Immune Checkpoint Blockade Immunotherapeutic Signature Optimizing Patient Selection and Treatment Strategies,” Small Methods 8 (2024): e2301685, 10.1002/smtd.202301685. 38546036

[21] M. S. Rooney , S. A. Shukla , C. J. Wu , G. Getz , and N. Hacohen , “Molecular and Genetic Properties of Tumors Associated With Local Immune Cytolytic Activity,” Cell 160 (2015): 48–61, 10.1016/j.cell.2014.12.033. 25594174 PMC4856474

[22] M. Ayers , J. Lunceford , M. Nebozhyn , et al., “IFN‐Gamma‐Related mRNA Profile Predicts Clinical Response to PD‐1 Blockade,” Journal of Clinical Investigation 127 (2017): 2930–2940, 10.1172/JCI91190. 28650338 PMC5531419

[23] W. Roh , P. L. Chen , A. Reuben , et al., “Integrated Molecular Analysis of Tumor Biopsies on Sequential CTLA‐4 and PD‐1 Blockade Reveals Markers of Response and Resistance,” Science Translational Medicine 9 (2017): eaah3560, 10.1126/scitranslmed.aah3560. 28251903 PMC5819607

[24] T. Davoli , H. Uno , E. C. Wooten , and S. J. Elledge , “Tumor Aneuploidy Correlates With Markers of Immune Evasion and With Reduced Response to Immunotherapy,” Science 355 (2017): eaaf8399, 10.1126/science.aaf8399. 28104840 PMC5592794

[25] L. Jerby‐Arnon , P. Shah , M. S. Cuoco , et al., “A Cancer Cell Program Promotes T Cell Exclusion and Resistance to Checkpoint Blockade,” Cell 175 (2018): 984–997.e924, 10.1016/j.cell.2018.09.006. 30388455 PMC6410377

[26] K. Yoshihara , M. Shahmoradgoli , E. Martínez , et al., “Inferring Tumour Purity and Stromal and Immune Cell Admixture From Expression Data,” Nature Communications 4 (2013): 2612, 10.1038/ncomms3612. PMC3826632 24113773

[27] S. Li , N. Zhang , H. Zhang , et al., “Deciphering the Role of LGALS2: Insights Into Tertiary Lymphoid Structure‐Associated Dendritic Cell Activation and Immunotherapeutic Potential in Breast Cancer Patients,” Molecular Cancer 23 (2024): 216, 10.1186/s12943-024-02126-4. 39350165 PMC11441145

[28] E. Becht , N. A. Giraldo , L. Lacroix , et al., “Estimating the Population Abundance of Tissue‐Infiltrating Immune and Stromal Cell Populations Using Gene Expression,” Genome Biology 17 (2016): 218, 10.1186/s13059-016-1070-5. 27765066 PMC5073889

[29] P. Charoentong , F. Finotello , M. Angelova , et al., “Pan‐Cancer Immunogenomic Analyses Reveal Genotype‐Immunophenotype Relationships and Predictors of Response to Checkpoint Blockade,” Cell Reports 18 (2017): 248–262, 10.1016/j.celrep.2016.12.019. 28052254

[30] Y. Fang , Y. Kong , G. Rong , Q. Luo , W. Liao , and D. Zeng , “Systematic Investigation of Tumor Microenvironment and Antitumor Immunity With IOBR,” Med Research 1 (2025): 136–140, 10.1002/mdr2.70001.

[31] D. S. Chen and I. Mellman , “Oncology Meets Immunology: The Cancer‐Immunity Cycle,” Immunity 39 (2013): 1–10, 10.1016/j.immuni.2013.07.012. 23890059

[32] V. Thorsson , D. L. Gibbs , S. D. Brown , et al., “The Immune Landscape of Cancer,” Immunity 48 (2018): 812–830.e814, 10.1016/j.immuni.2018.03.023. 29628290 PMC5982584

[33] N. Zhang , H. Zhang , S. Li , et al., “Uncovering the Predictive and Immunomodulatory Potential of Transient Receptor Potential Melastatin Family‐Related CCNE1 in Pan‐Cancer,” Molecular Cancer 23 (2024): 258, 10.1186/s12943-024-02169-7. 39551726 PMC11572178

[34] Y. Zhou , B. Zhou , L. Pache , et al., “Metascape Provides a Biologist‐Oriented Resource for the Analysis of Systems‐Level Datasets,” Nature Communications 10 (2019): 1523, 10.1038/s41467-019-09234-6. PMC6447622 30944313

[35] D. Maeser , R. F. Gruener , and R. S. Huang , “oncoPredict: An R Package for Predicting In Vivo or Cancer Patient Drug Response and Biomarkers From Cell Line Screening Data,” Briefings in Bioinformatics 22 (2021): bbab260 10.1093/bib/bbab260. 34260682 PMC8574972

[36] C. H. Mermel , S. E. Schumacher , B. Hill , M. L. Meyerson , R. Beroukhim , and G. Getz , “GISTIC2.0 Facilitates Sensitive and Confident Localization of the Targets of Focal Somatic Copy‐Number Alteration in Human Cancers,” Genome Biology 12 (2011): R41, 10.1186/gb-2011-12-4-r41. 21527027 PMC3218867

[37] K. Trpkov , S. R. Williamson , Y. Gao , et al., “Low‐Grade Oncocytic Tumour of Kidney (CD117‐Negative, Cytokeratin 7‐Positive): A Distinct Entity?,” Histopathology 75 (2019): 174–184, 10.1111/his.13865. 30895640

[38] F. Al‐Obeidat , A. Rashid , W. Hafez , et al., “The Accuracy of Artificial Intelligence in the Diagnosis of Soft Tissue Sarcoma: A Systematic Review and Meta‐Analysis,” Current Problems in Surgery 66 (2025): 101743, 10.1016/j.cpsurg.2025.101743. 40306879

[39] D. A. Hla and D. I. Hindin , “Generative AI & Machine Learning in Surgical Education,” Current Problems in Surgery 63 (2025): 101701, 10.1016/j.cpsurg.2024.101701. 39922636

[40] R. A. C. Skov , J. Lawaetz , M. Strøm , et al., “Machine Learning Enhances Assessment of Proficiency in Endovascular Aortic Repair Simulations,” Current Problems in Surgery 61 (2024): 101576, 10.1016/j.cpsurg.2024.101576. 39266132

[41] C. Y. Chen , J. J. Wu , Y. J. Lin , et al., “Significance of Hypermethylation of Tumor‐Suppressor Genes PTGER4 and ZNF43 at CpG Sites in the Prognosis of Colorectal Cancer,” International Journal of Molecular Sciences 23 (2022): 10225, 10.3390/ijms231810225. 36142151 PMC9499344

[42] S. Yu , R. Tu , Z. Chen , et al., “Association of PTGER4 and PRKAA1 Genetic Polymorphisms With Gastric Cancer,” BMC Medical Genomics 16 (2023): 209, 10.1186/s12920-023-01645-1. 37670284 PMC10478487

[43] J. Gault , I. Liko , M. Landreh , et al., “Combining Native and ‘omics’ Mass Spectrometry to Identify Endogenous Ligands Bound to Membrane Proteins,” Nature Methods 17 (2020): 505–508, 10.1038/s41592-020-0821-0. 32371966 PMC7332344

